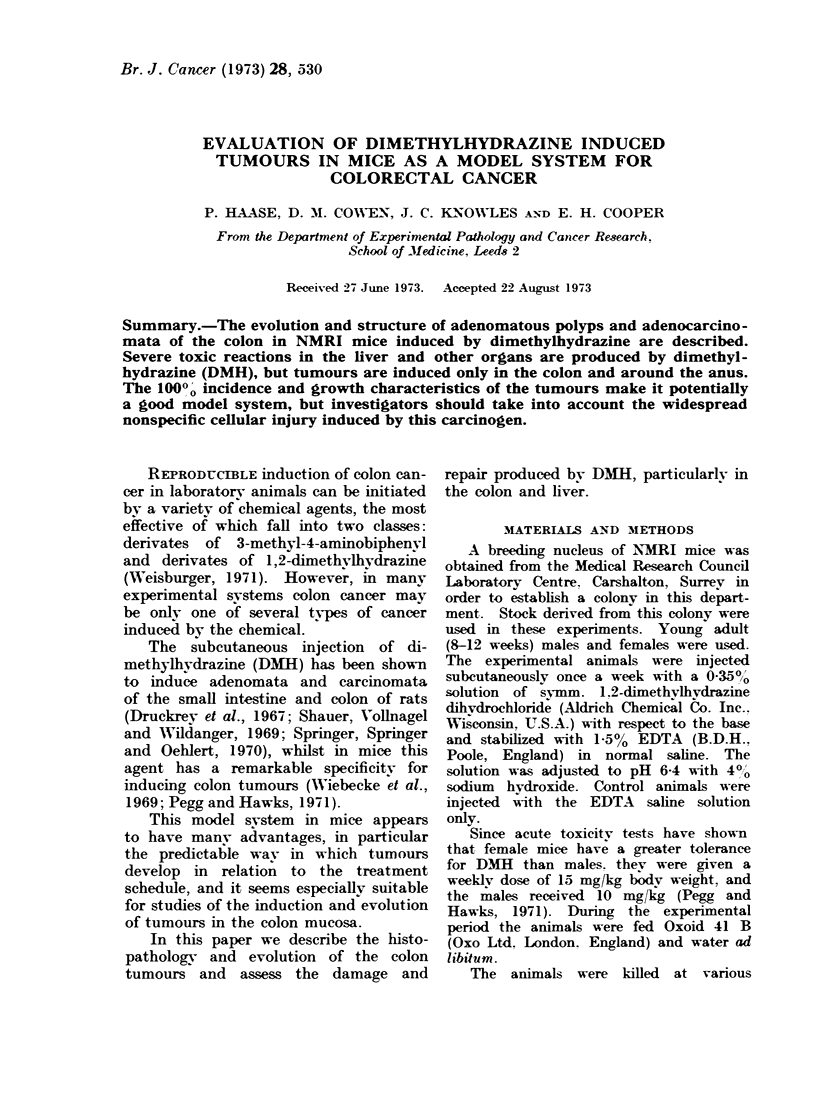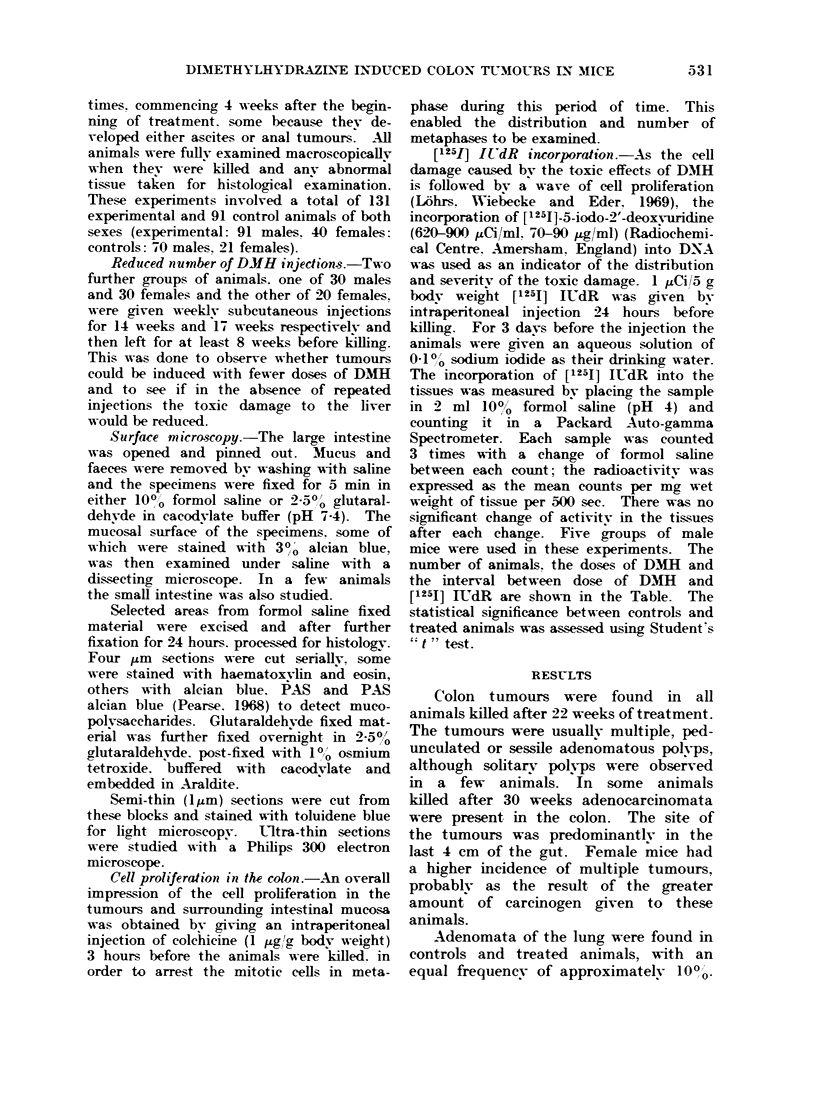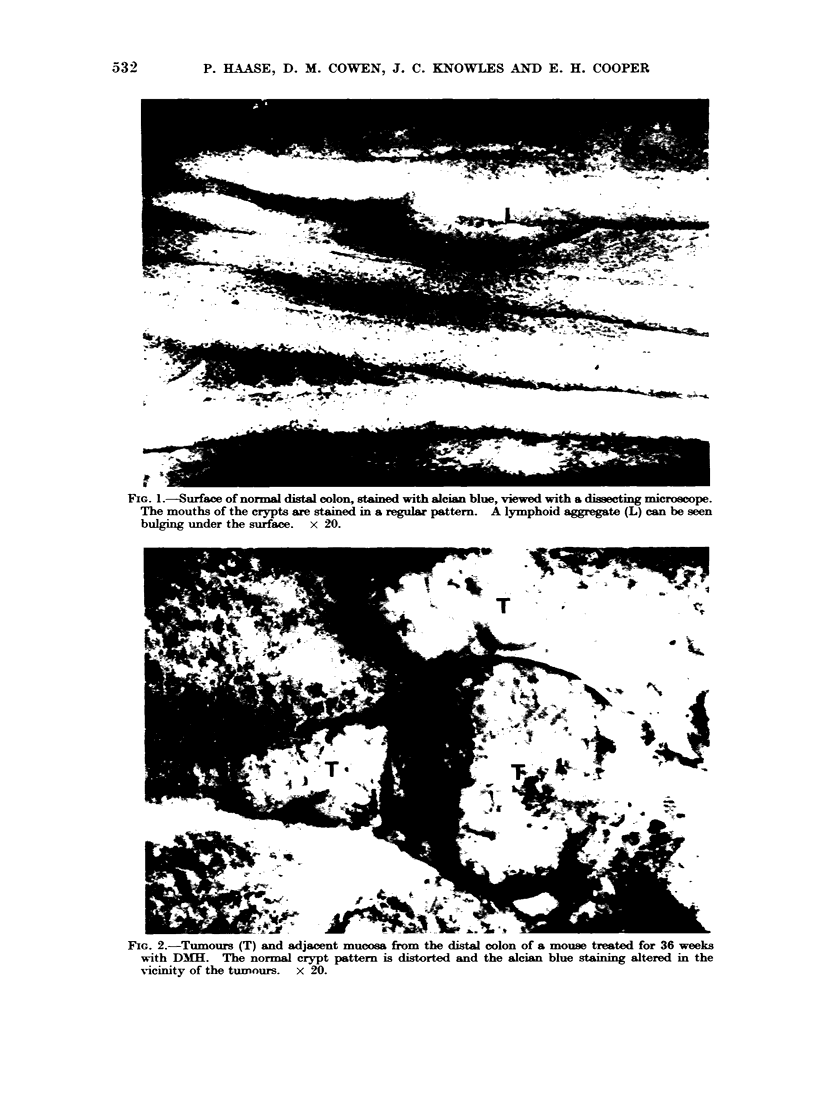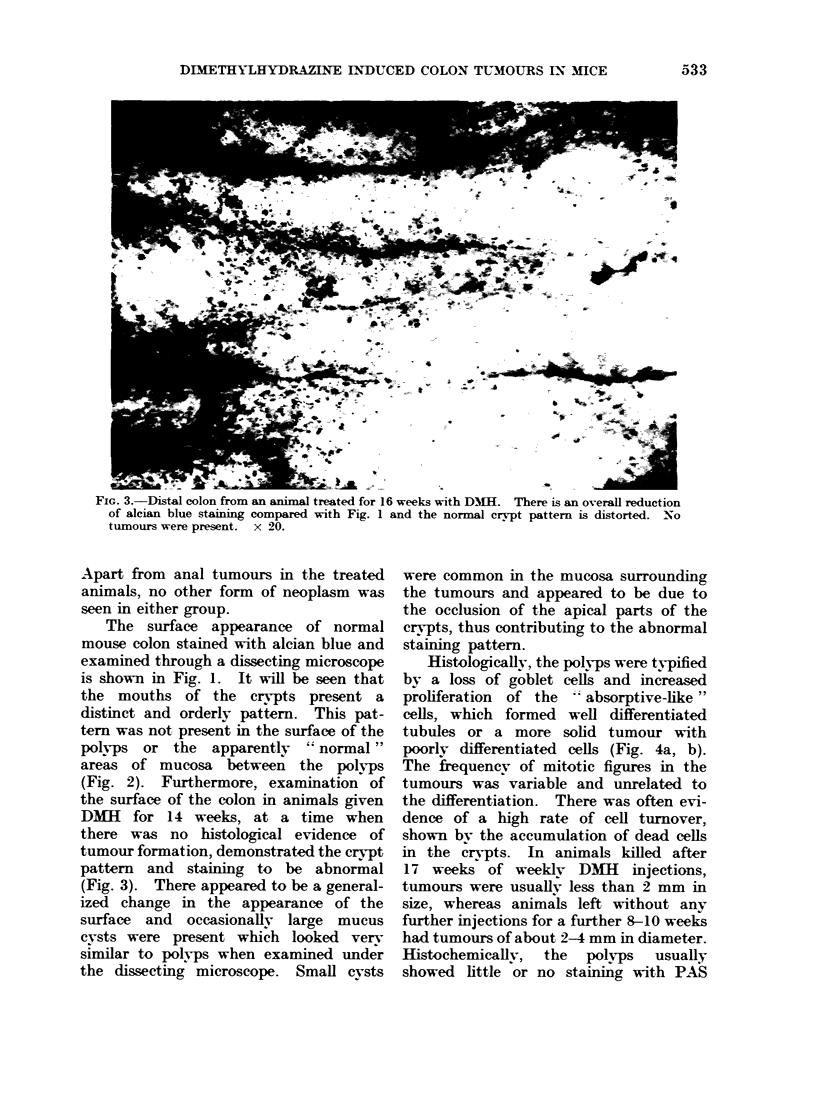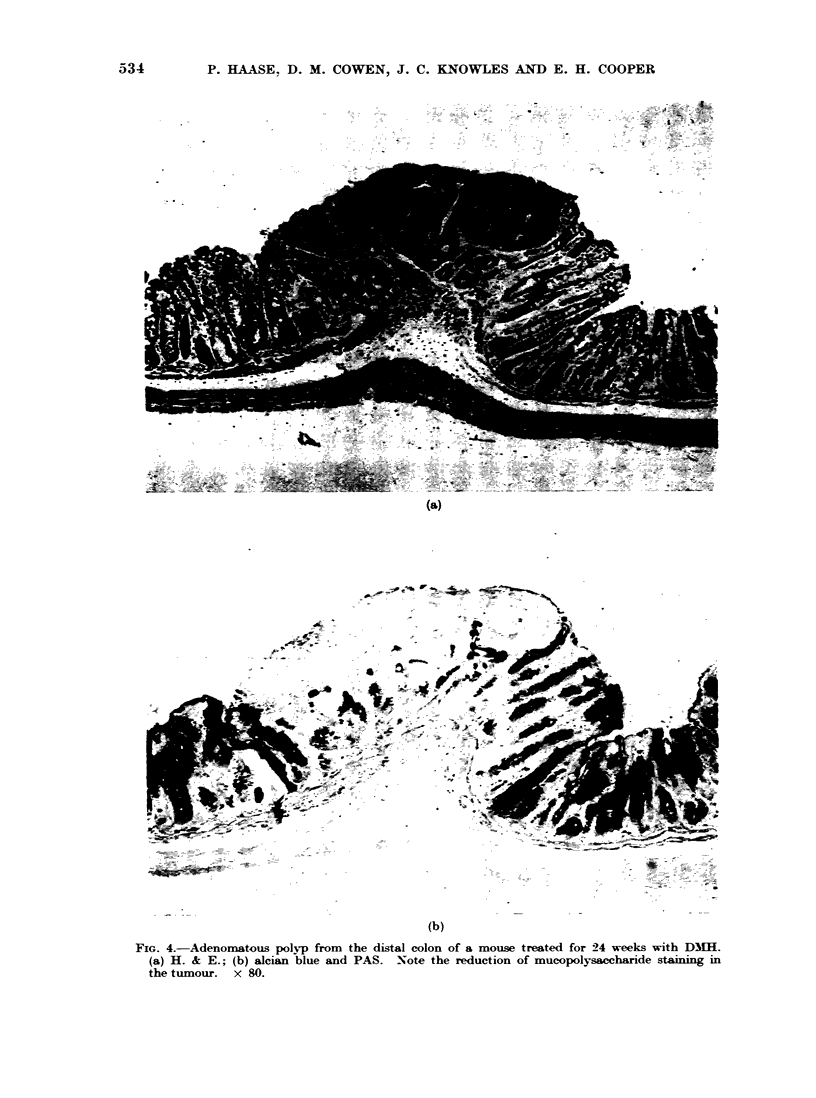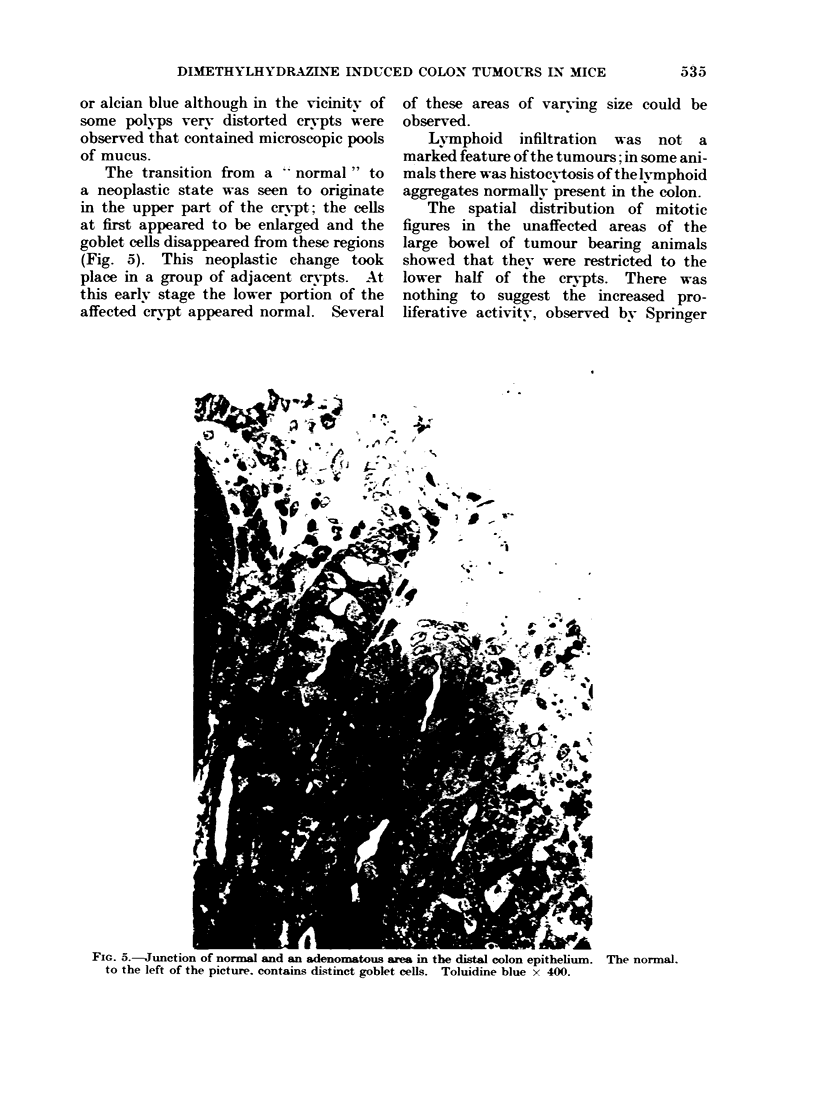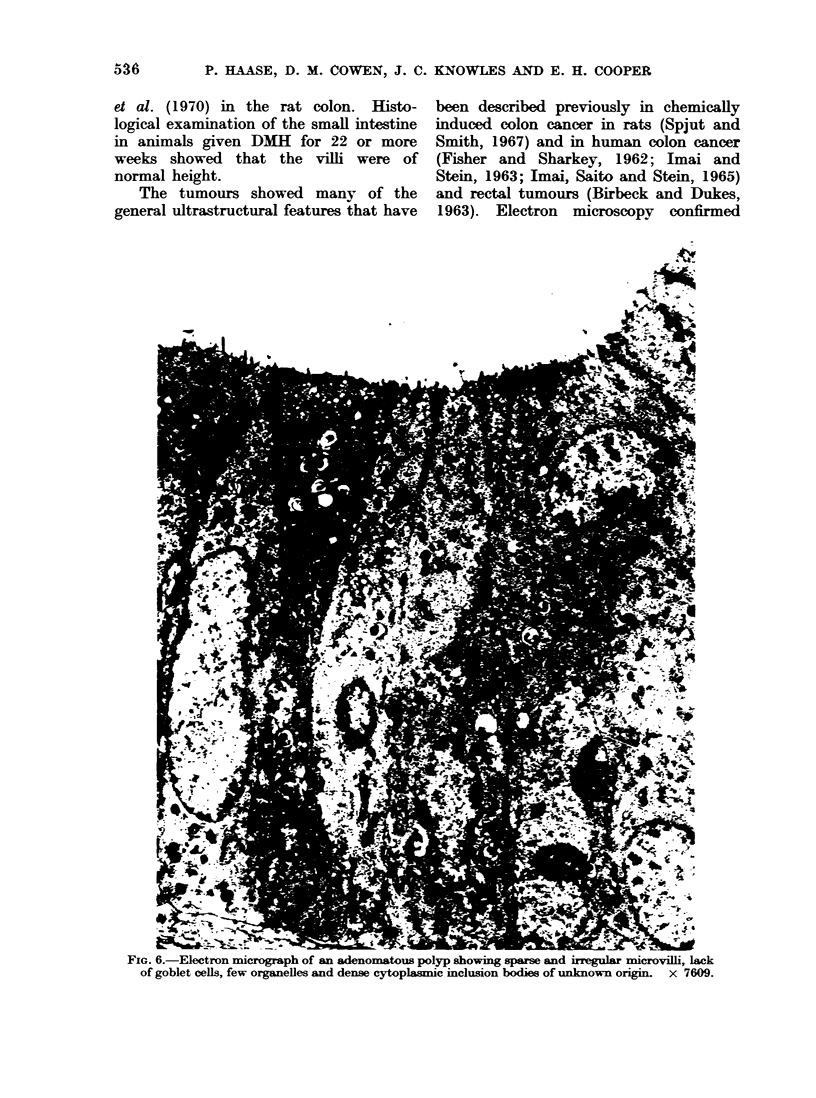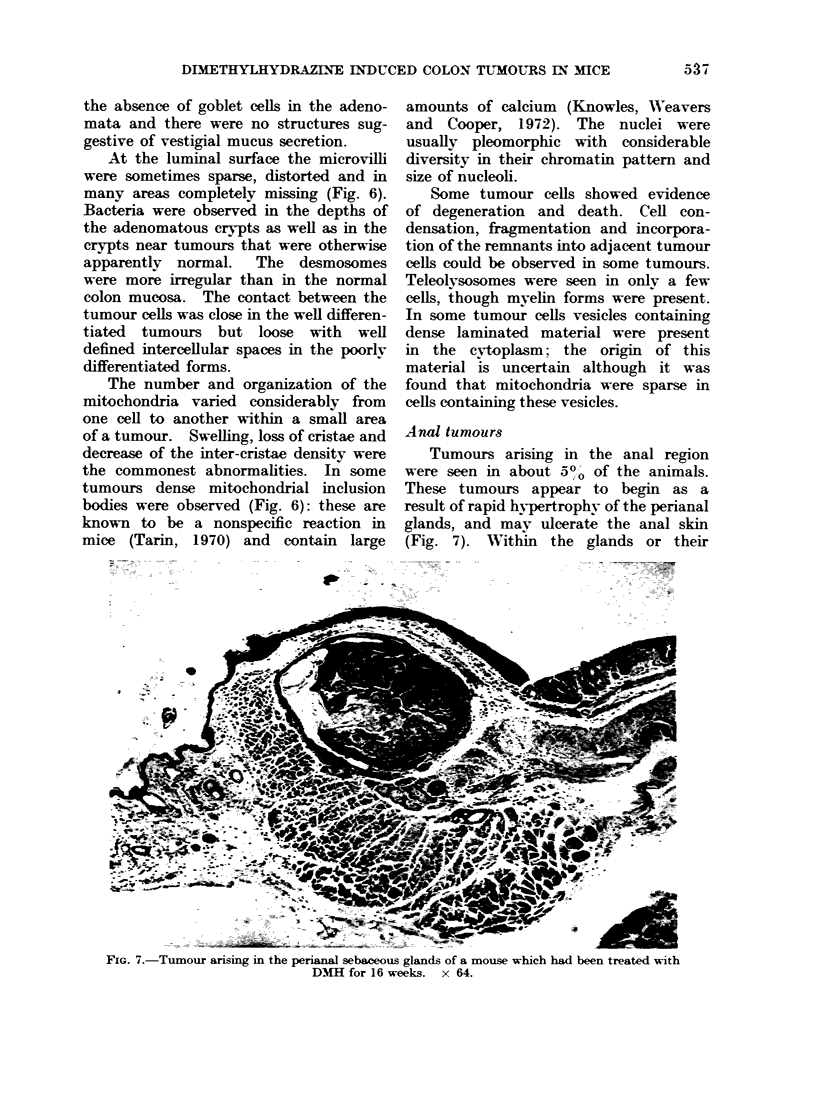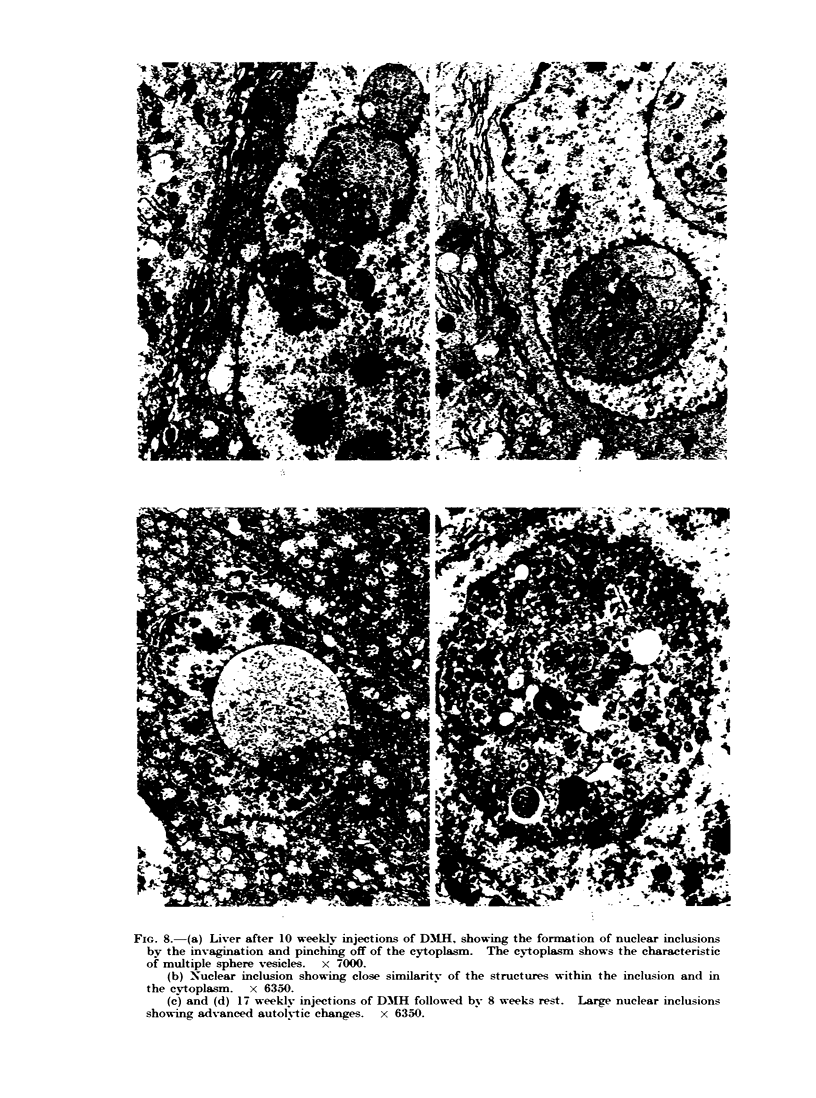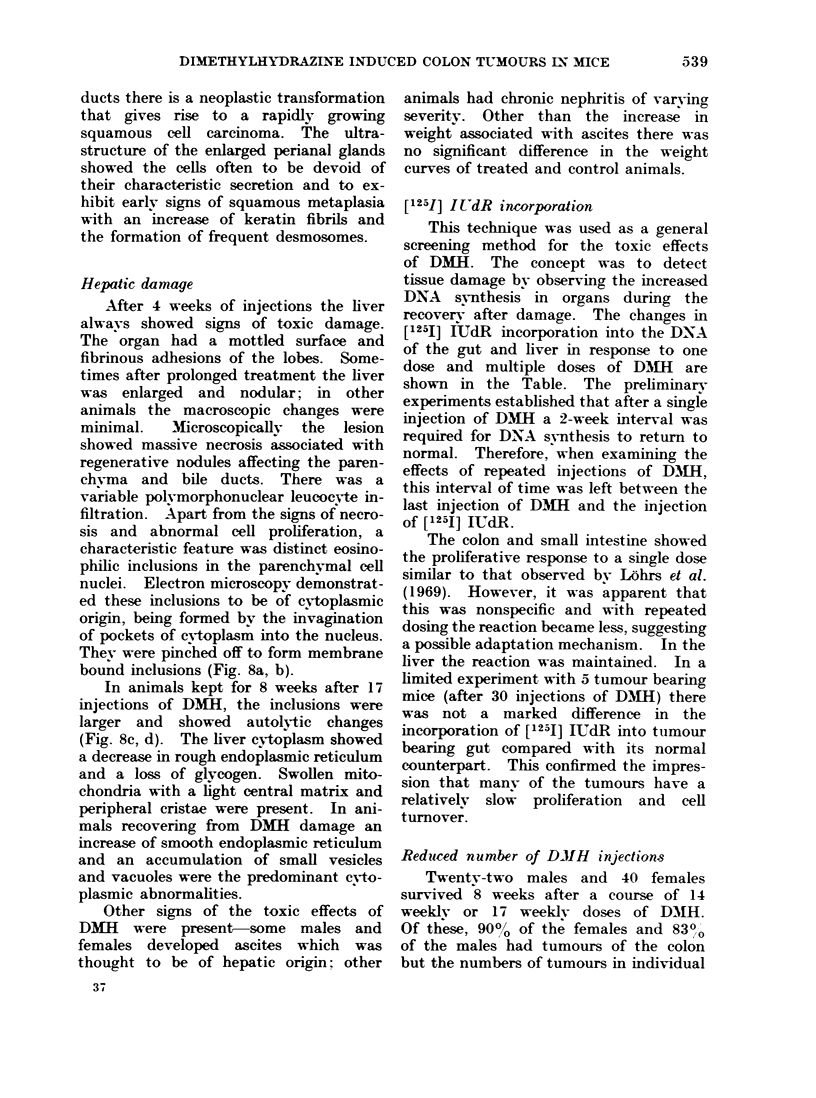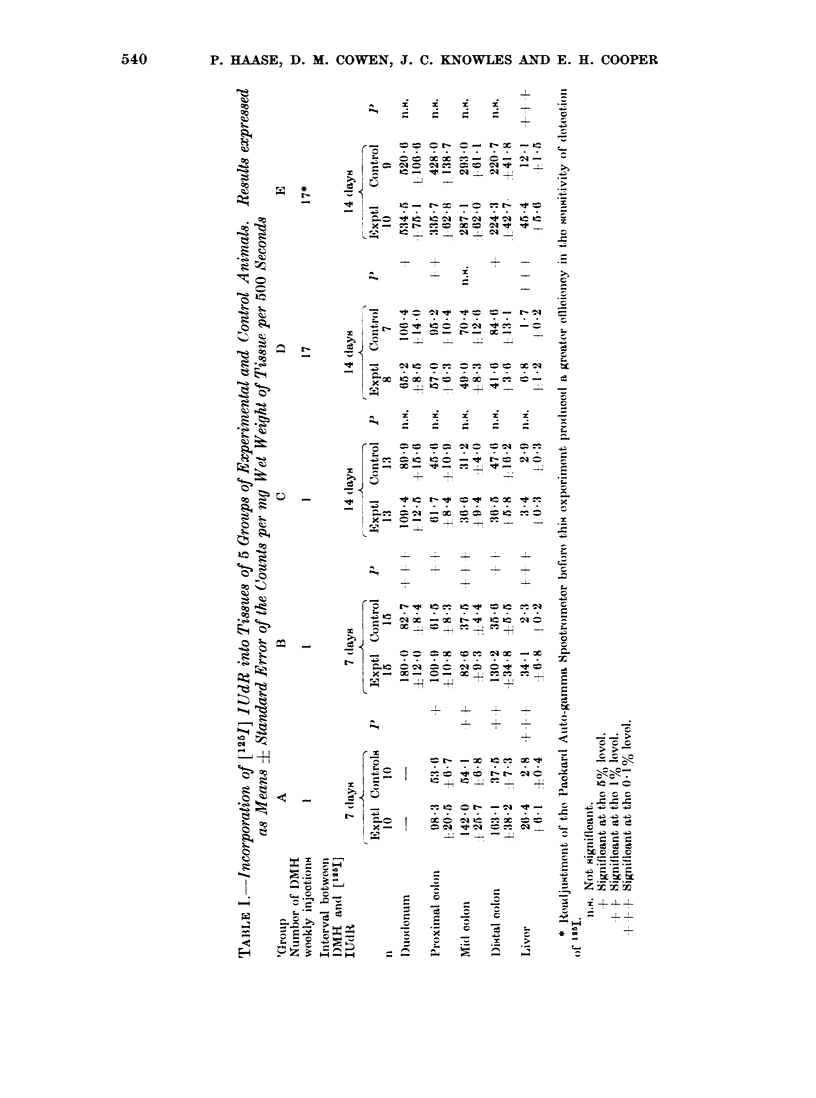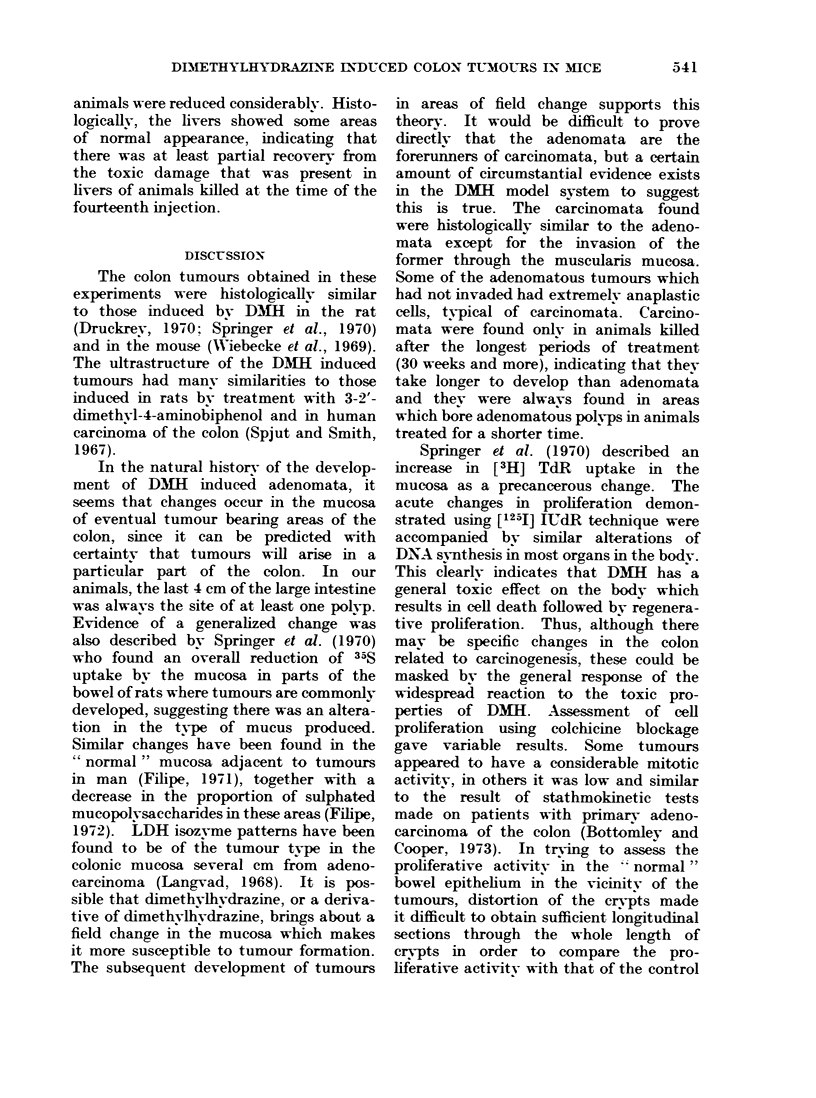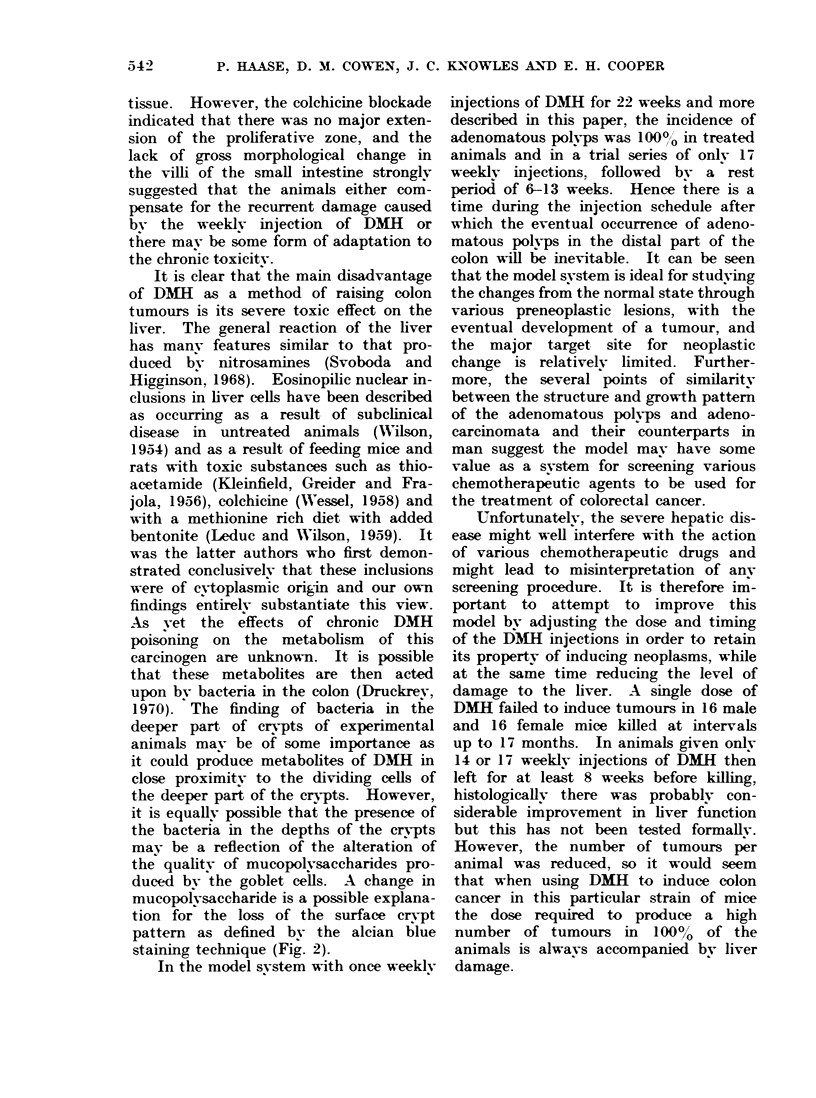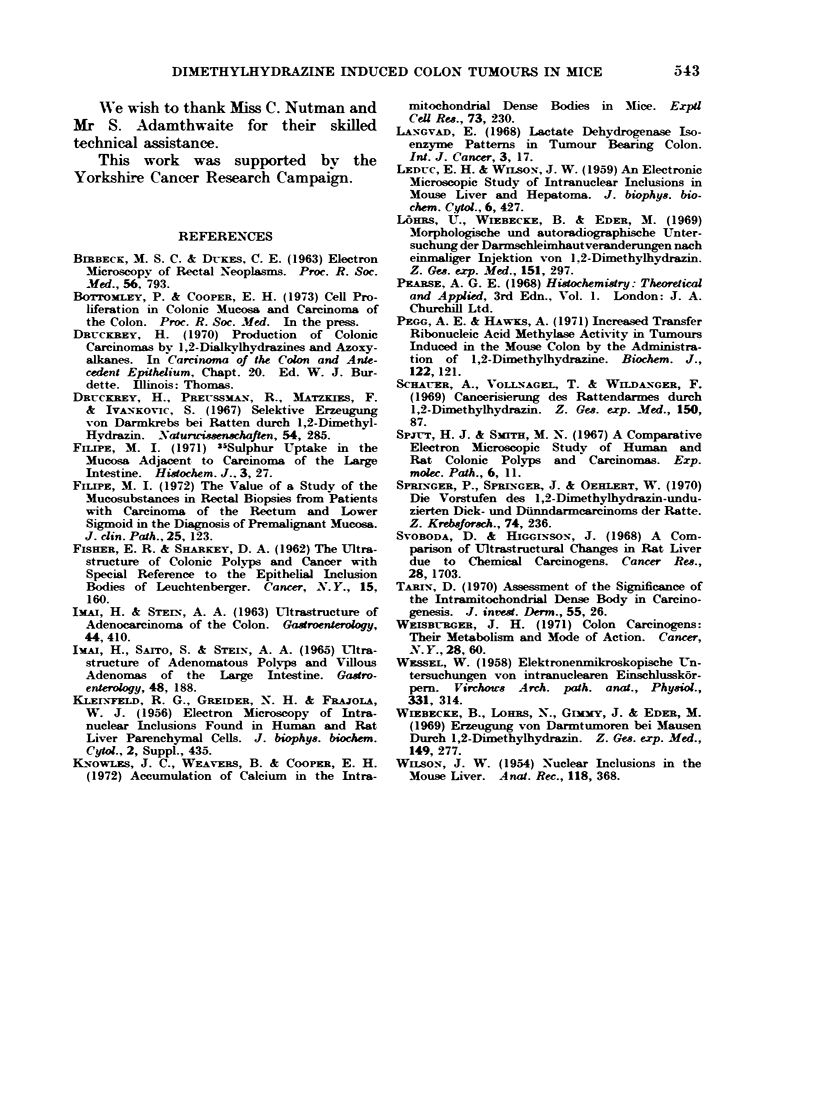# Evaluation of Dimethylhydrazine Induced Tumours in Mice as a Model System for Colorectal Cancer

**DOI:** 10.1038/bjc.1973.183

**Published:** 1973-12

**Authors:** P. Haase, D. M. Cowen, J. C. Knowles, E. H. Cooper

## Abstract

**Images:**


Br. J. Cancer (1973) **28**, 530

### EVALUATION OF DIMETHYLHYDRAZINE INDUCED TUMOURS IN MICE AS A MODEL SYSTEM FOR COLORECTAL CANCER

P. HAASE, D. M. COWEN, J. C. KNOWLES AND E. H. COOPER

*From the Department of Experimental Pathology and Cancer Research, School of Medicine, Leeds 2*

Received 27 June 1973. Accepted 22 August 1973

**Summary.—The evolution and structure of adenomatous polyps and adenocarcinomata of the colon in NMRI mice induced by dimethylhydrazine are described. Severe toxic reactions in the liver and other organs are produced by dimethylhydrazine (DMH), but tumours are induced only in the colon and around the anus. The 100% incidence and growth characteristics of the tumours make it potentially a good model system, but investigators should take into account the widespread nonspecific cellular injury induced by this carcinogen.**

REPRODUCIBLE induction of colon cancer in laboratory animals can be initiated by a variety of chemical agents, the most effective of which fall into two classes: derivates of 3-methyl-4-aminobiphenyl and derivates of 1,2-dimethylhydrazine (Weisburger, 1971). However, in many experimental systems colon cancer may be only one of several types of cancer induced by the chemical.

The subcutaneous injection of dimethylhydrazine (DMH) has been shown to induce adenomata and carcinomata of the small intestine and colon of rats (Druckrey *et al.*, 1967; Shauer, Vollnagel and Wildanger, 1969; Springer, Springer and Oehlert, 1970), whilst in mice this agent has a remarkable specificity for inducing colon tumours (Wiebecke *et al.*, 1969; Pegg and Hawks, 1971).

This model system in mice appears to have many advantages, in particular the predictable way in which tumours develop in relation to the treatment schedule, and it seems especially suitable for studies of the induction and evolution of tumours in the colon mucosa.

In this paper we describe the histopathology and evolution of the colon tumours and assess the damage and repair produced by DMH, particularly in the colon and liver.

### MATERIALS AND METHODS

A breeding nucleus of NMRI mice was obtained from the Medical Research Council Laboratory Centre, Carshalton, Surrey in order to establish a colony in this department. Stock derived from this colony were used in these experiments. Young adult (8–12 weeks) males and females were used. The experimental animals were injected subcutaneously once a week with a 0·35% solution of symm. 1,2-dimethylhydrazine dihydrochloride (Aldrich Chemical Co. Inc., Wisconsin, U.S.A.) with respect to the base and stabilized with 1·5% EDTA (B.D.H., Poole, England) in normal saline. The solution was adjusted to pH 6·4 with 4% sodium hydroxide. Control animals were injected with the EDTA saline solution only.

Since acute toxicity tests have shown that female mice have a greater tolerance for DMH than males, they were given a weekly dose of 15 mg/kg body weight, and the males received 10 mg/kg (Pegg and Hawks, 1971). During the experimental period the animals were fed Oxoid 41 B (Oxo Ltd, London, England) and water *ad libitum*.

The animals were killed at various times, commencing 4 weeks after the beginning of treatment, some because they developed either ascites or anal tumours. All animals were fully examined macroscopically when they were killed and any abnormal tissue taken for histological examination. These experiments involved a total of 131 experimental and 91 control animals of both sexes (experimental: 91 males, 40 females; controls: 70 males, 21 females).

*Reduced number of DMH injections.*—Two further groups of animals, one of 30 males and 30 females and the other of 20 females, were given weekly subcutaneous injections for 14 weeks and 17 weeks respectively and then left for at least 8 weeks before killing. This was done to observe whether tumours could be induced with fewer doses of DMH and to see if in the absence of repeated injections the toxic damage to the liver would be reduced.

*Surface microscopy.*—The large intestine was opened and pinned out. Mucus and faeces were removed by washing with saline and the specimens were fixed for 5 min in either 10% formol saline or 2·5% glutaraldehyde in cacodylate buffer (pH 7·4). The mucosal surface of the specimens, some of which were stained with 3% alcian blue, was then examined under saline with a dissecting microscope. In a few animals the small intestine was also studied.

Selected areas from formol saline fixed material were excised and after further fixation for 24 hours, processed for histology. Four $\mu$m sections were cut serially, some were stained with haematoxylin and eosin, others with alcian blue, PAS and PAS alcian blue (Pearse, 1968) to detect mucopolysaccharides. Glutaraldehyde fixed material was further fixed overnight in 2·5% glutaraldehyde, post-fixed with 1% osmium tetroxide, buffered with cacodylate and embedded in Araldite.

Semi-thin (1$\mu$m) sections were cut from these blocks and stained with toluidene blue for light microscopy. Ultra-thin sections were studied with a Philips 300 electron microscope.

*Cell proliferation in the colon.*—An overall impression of the cell proliferation in the tumours and surrounding intestinal mucosa was obtained by giving an intraperitoneal injection of colchicine (1 $\mu$g/g body weight) 3 hours before the animals were killed, in order to arrest the mitotic cells in metaphase during this period of time. This enabled the distribution and number of metaphases to be examined.

*[$^{125}$I] IUdR incorporation.*—As the cell damage caused by the toxic effects of DMH is followed by a wave of cell proliferation (Löhrs, Wiebecke and Eder, 1969), the incorporation of [$^{125}$I]-5-iodo-2′-deoxyuridine (620–900 $\mu$Ci/ml, 70–90 $\mu$g/ml) (Radiochemical Centre, Amersham, England) into DNA was used as an indicator of the distribution and severity of the toxic damage. 1 $\mu$Ci/5 g body weight [$^{125}$I] IUdR was given by intraperitoneal injection 24 hours before killing. For 3 days before the injection the animals were given an aqueous solution of 0·1% sodium iodide as their drinking water. The incorporation of [$^{125}$I] IUdR into the tissues was measured by placing the sample in 2 ml 10% formol saline (pH 4) and counting it in a Packard Auto-gamma Spectrometer. Each sample was counted 3 times with a change of formol saline between each count; the radioactivity was expressed as the mean counts per mg wet weight of tissue per 500 sec. There was no significant change of activity in the tissues after each change. Five groups of male mice were used in these experiments. The number of animals, the doses of DMH and the interval between dose of DMH and [$^{125}$I] IUdR are shown in the Table. The statistical significance between controls and treated animals was assessed using Student's "*t*" test.

### RESULTS

Colon tumours were found in all animals killed after 22 weeks of treatment. The tumours were usually multiple, pedunculated or sessile adenomatous polyps, although solitary polyps were observed in a few animals. In some animals killed after 30 weeks adenocarcinomata were present in the colon. The site of the tumours was predominantly in the last 4 cm of the gut. Female mice had a higher incidence of multiple tumours, probably as the result of the greater amount of carcinogen given to these animals.

Adenomata of the lung were found in controls and treated animals, with an equal frequency of approximately 10%.

FIG. 1.—Surface of normal distal colon, stained with alcian blue, viewed with a dissecting microscope. The mouths of the crypts are stained in a regular pattern. A lymphoid aggregate (L) can be seen bulging under the surface. × 20.

FIG. 2.—Tumours (T) and adjacent mucosa from the distal colon of a mouse treated for 36 weeks with DMH. The normal crypt pattern is distorted and the alcian blue staining altered in the vicinity of the tumours. × 20.

FIG. 3.—Distal colon from an animal treated for 16 weeks with DMH. There is an overall reduction of alcian blue staining compared with Fig. 1 and the normal crypt pattern is distorted. No tumours were present. × 20.

Apart from anal tumours in the treated animals, no other form of neoplasm was seen in either group.

The surface appearance of normal mouse colon stained with alcian blue and examined through a dissecting microscope is shown in Fig. 1. It will be seen that the mouths of the crypts present a distinct and orderly pattern. This pattern was not present in the surface of the polyps or the apparently "normal" areas of mucosa between the polyps (Fig. 2). Furthermore, examination of the surface of the colon in animals given DMH for 14 weeks, at a time when there was no histological evidence of tumour formation, demonstrated the crypt pattern and staining to be abnormal (Fig. 3). There appeared to be a generalized change in the appearance of the surface and occasionally large mucus cysts were present which looked very similar to polyps when examined under the dissecting microscope. Small cysts were common in the mucosa surrounding the tumours and appeared to be due to the occlusion of the apical parts of the crypts, thus contributing to the abnormal staining pattern.

Histologically, the polyps were typified by a loss of goblet cells and increased proliferation of the "absorptive-like" cells, which formed well differentiated tubules or a more solid tumour with poorly differentiated cells (Fig. 4a, b). The frequency of mitotic figures in the tumours was variable and unrelated to the differentiation. There was often evidence of a high rate of cell turnover, shown by the accumulation of dead cells in the crypts. In animals killed after 17 weeks of weekly DMH injections, tumours were usually less than 2 mm in size, whereas animals left without any further injections for a further 8–10 weeks had tumours of about 2–4 mm in diameter. Histochemically, the polyps usually showed little or no staining with PAS

FIG. 4.—Adenomatous polyp from the distal colon of a mouse treated for 24 weeks with DMH. (a) H. & E.; (b) alcian blue and PAS. Note the reduction of mucopolysaccharide staining in the tumour. × 80.

or alcian blue although in the vicinity of some polyps very distorted crypts were observed that contained microscopic pools of mucus.

The transition from a " normal " to a neoplastic state was seen to originate in the upper part of the crypt; the cells at first appeared to be enlarged and the goblet cells disappeared from these regions (Fig. 5). This neoplastic change took place in a group of adjacent crypts. At this early stage the lower portion of the affected crypt appeared normal. Several of these areas of varying size could be observed.

Lymphoid infiltration was not a marked feature of the tumours; in some animals there was histocytosis of the lymphoid aggregates normally present in the colon.

The spatial distribution of mitotic figures in the unaffected areas of the large bowel of tumour bearing animals showed that they were restricted to the lower half of the crypts. There was nothing to suggest the increased proliferative activity, observed by Springer

FIG. 5.—Junction of normal and an adenomatous area in the distal colon epithelium. The normal, to the left of the picture, contains distinct goblet cells. Toluidine blue × 400.

*et al.* (1970) in the rat colon. Histological examination of the small intestine in animals given DMH for 22 or more weeks showed that the villi were of normal height.

The tumours showed many of the general ultrastructural features that have been described previously in chemically induced colon cancer in rats (Spjut and Smith, 1967) and in human colon cancer (Fisher and Sharkey, 1962; Imai and Stein, 1963; Imai, Saito and Stein, 1965) and rectal tumours (Birbeck and Dukes, 1963). Electron microscopy confirmed

FIG. 6.—Electron micrograph of an adenomatous polyp showing sparse and irregular microvilli, lack of goblet cells, few organelles and dense cytoplasmic inclusion bodies of unknown origin. × 7609.

the absence of goblet cells in the adenomata and there were no structures suggestive of vestigial mucus secretion.

At the luminal surface the microvilli were sometimes sparse, distorted and in many areas completely missing (Fig. 6). Bacteria were observed in the depths of the adenomatous crypts as well as in the crypts near tumours that were otherwise apparently normal. The desmosomes were more irregular than in the normal colon mucosa. The contact between the tumour cells was close in the well differentiated tumours but loose with well defined intercellular spaces in the poorly differentiated forms.

The number and organization of the mitochondria varied considerably from one cell to another within a small area of a tumour. Swelling, loss of cristae and decrease of the inter-cristae density were the commonest abnormalities. In some tumours dense mitochondrial inclusion bodies were observed (Fig. 6): these are known to be a nonspecific reaction in mice (Tarin, 1970) and contain large amounts of calcium (Knowles, Weavers and Cooper, 1972). The nuclei were usually pleomorphic with considerable diversity in their chromatin pattern and size of nucleoli.

Some tumour cells showed evidence of degeneration and death. Cell condensation, fragmentation and incorporation of the remnants into adjacent tumour cells could be observed in some tumours. Teleolysosomes were seen in only a few cells, though myelin forms were present. In some tumour cells vesicles containing dense laminated material were present in the cytoplasm; the origin of this material is uncertain although it was found that mitochondria were sparse in cells containing these vesicles.

*Anal tumours*

Tumours arising in the anal region were seen in about 5% of the animals. These tumours appear to begin as a result of rapid hypertrophy of the perianal glands, and may ulcerate the anal skin (Fig. 7). Within the glands or their

FIG. 7.—Tumour arising in the perianal sebaceous glands of a mouse which had been treated with DMH for 16 weeks. × 64.

Fig. 8.—(a) Liver after 10 weekly injections of DMH, showing the formation of nuclear inclusions by the invagination and pinching off of the cytoplasm. The cytoplasm shows the characteristic of multiple sphere vesicles. × 7000.

(b) Nuclear inclusion showing close similarity of the structures within the inclusion and in the cytoplasm. × 6350.

(c) and (d) 17 weekly injections of DMH followed by 8 weeks rest. Large nuclear inclusions showing advanced autolytic changes. × 6350.

ducts there is a neoplastic transformation that gives rise to a rapidly growing squamous cell carcinoma. The ultrastructure of the enlarged perianal glands showed the cells often to be devoid of their characteristic secretion and to exhibit early signs of squamous metaplasia with an increase of keratin fibrils and the formation of frequent desmosomes.

*Hepatic damage*

After 4 weeks of injections the liver always showed signs of toxic damage. The organ had a mottled surface and fibrinous adhesions of the lobes. Sometimes after prolonged treatment the liver was enlarged and nodular; in other animals the macroscopic changes were minimal. Microscopically the lesion showed massive necrosis associated with regenerative nodules affecting the parenchyma and bile ducts. There was a variable polymorphonuclear leucocyte infiltration. Apart from the signs of necrosis and abnormal cell proliferation, a characteristic feature was distinct eosinophilic inclusions in the parenchymal cell nuclei. Electron microscopy demonstrated these inclusions to be of cytoplasmic origin, being formed by the invagination of pockets of cytoplasm into the nucleus. They were pinched off to form membrane bound inclusions (Fig. 8a, b).

In animals kept for 8 weeks after 17 injections of DMH, the inclusions were larger and showed autolytic changes (Fig. 8c, d). The liver cytoplasm showed a decrease in rough endoplasmic reticulum and a loss of glycogen. Swollen mitochondria with a light central matrix and peripheral cristae were present. In animals recovering from DMH damage an increase of smooth endoplasmic reticulum and an accumulation of small vesicles and vacuoles were the predominant cytoplasmic abnormalities.

Other signs of the toxic effects of DMH were present—some males and females developed ascites which was thought to be of hepatic origin; other animals had chronic nephritis of varying severity. Other than the increase in weight associated with ascites there was no significant difference in the weight curves of treated and control animals.

*[$^{125}$I] IUdR incorporation*

This technique was used as a general screening method for the toxic effects of DMH. The concept was to detect tissue damage by observing the increased DNA synthesis in organs during the recovery after damage. The changes in [$^{125}$I] IUdR incorporation into the DNA of the gut and liver in response to one dose and multiple doses of DMH are shown in the Table. The preliminary experiments established that after a single injection of DMH a 2-week interval was required for DNA synthesis to return to normal. Therefore, when examining the effects of repeated injections of DMH, this interval of time was left between the last injection of DMH and the injection of [$^{125}$I] IUdR.

The colon and small intestine showed the proliferative response to a single dose similar to that observed by Löhrs *et al.* (1969). However, it was apparent that this was nonspecific and with repeated dosing the reaction became less, suggesting a possible adaptation mechanism. In the liver the reaction was maintained. In a limited experiment with 5 tumour bearing mice (after 30 injections of DMH) there was not a marked difference in the incorporation of [$^{125}$I] IUdR into tumour bearing gut compared with its normal counterpart. This confirmed the impression that many of the tumours have a relatively slow proliferation and cell turnover.

*Reduced number of DMH injections*

Twenty-two males and 40 females survived 8 weeks after a course of 14 weekly or 17 weekly doses of DMH. Of these, 90% of the females and 83% of the males had tumours of the colon but the numbers of tumours in individual

TABLE I.—*Incorporation of [125I] IUdR into Tissues of 5 Groups of Experimental and Control Animals. Results expressed as Means ± Standard Error of the Counts per mg Wet Weight of Tissue per 500 Seconds*

| Group | A | | | B | | | C | | | D | | | E | | |
|---|---|---|---|---|---|---|---|---|---|---|---|---|---|---|---|
| Number of DMH weekly injections | 1 | | | 1 | | | 1 | | | 17 | | | 17* | | |
| Interval between DMH and [125I] IUdR | 7 days | | | 7 days | | | 14 days | | | 14 days | | | 14 days | | |
| | Exptl | Controls | P | Exptl | Control | P | Exptl | Control | P | Exptl | Control | P | Exptl | Control | P |
| n | 10 | 10 | | 15 | 15 | | 13 | 13 | | 8 | 7 | | 10 | 9 | |
| Duodenum | — | — | | 180·0 ±12·0 | 82·7 ±8·4 | + + + | 109·4 ±12·5 | 89·9 ±15·6 | n.s. | 65·2 ±8·5 | 106·4 ±14·0 | + | 534·5 ±75·1 | 520·6 ±106·6 | n.s. |
| Proximal colon | 98·3 ±20·5 | 53·6 ±6·7 | + | 109·9 ±10·8 | 61·5 ±8·3 | + + | 61·7 ±8·4 | 45·6 ±10·9 | n.s. | 57·0 ±6·3 | 95·2 ±10·4 | + + | 335·7 ±62·8 | 428·0 ±138·7 | n.s. |
| Mid colon | 142·0 ±25·7 | 54·1 ±6·8 | + + | 82·6 ±9·3 | 37·5 ±4·4 | + + + | 36·6 ±9·4 | 31·2 ±4·0 | n.s. | 49·0 ±8·3 | 70·4 ±12·6 | n.s. | 287·1 ±62·0 | 293·0 ±61·1 | n.s. |
| Distal colon | 163·1 ±38·2 | 37·5 ±7·3 | + + | 130·2 ±34·8 | 35·6 ±5·5 | + + | 36·5 ±5·8 | 47·6 ±16·2 | n.s. | 41·6 ±3·6 | 84·6 ±13·1 | + | 224·3 ±42·7 | 220·7 ±41·8 | n.s. |
| Liver | 26·4 ±6·1 | 2·8 ±0·4 | + + + | 34·1 ±6·8 | 2·3 ±0·2 | + + + | 3·4 ±0·3 | 2·9 ±0·3 | n.s. | 6·8 ±1·2 | 1·7 ±0·2 | + + + | 45·4 ±5·6 | 12·1 ±1·5 | + + + |

* Readjustment of the Packard Auto-gamma Spectrometer before this experiment produced a greater efficiency in the sensitivity of detection of 125I.

n.s. Not significant.

\+ Significant at the 5% level.

\+ + Significant at the 1% level.

\+ + + Significant at the 0·1% level.

animals were reduced considerably. Histologically, the livers showed some areas of normal appearance, indicating that there was at least partial recovery from the toxic damage that was present in livers of animals killed at the time of the fourteenth injection.

### DISCUSSION

The colon tumours obtained in these experiments were histologically similar to those induced by DMH in the rat (Druckrey, 1970; Springer *et al.*, 1970) and in the mouse (Wiebecke *et al.*, 1969). The ultrastructure of the DMH induced tumours had many similarities to those induced in rats by treatment with 3-2′-dimethyl-4-aminobiphenol and in human carcinoma of the colon (Spjut and Smith, 1967).

In the natural history of the development of DMH induced adenomata, it seems that changes occur in the mucosa of eventual tumour bearing areas of the colon, since it can be predicted with certainty that tumours will arise in a particular part of the colon. In our animals, the last 4 cm of the large intestine was always the site of at least one polyp. Evidence of a generalized change was also described by Springer *et al.* (1970) who found an overall reduction of $^{35}S$ uptake by the mucosa in parts of the bowel of rats where tumours are commonly developed, suggesting there was an alteration in the type of mucus produced. Similar changes have been found in the " normal " mucosa adjacent to tumours in man (Filipe, 1971), together with a decrease in the proportion of sulphated mucopolysaccharides in these areas (Filipe, 1972). LDH isozyme patterns have been found to be of the tumour type in the colonic mucosa several cm from adenocarcinoma (Langvad, 1968). It is possible that dimethylhydrazine, or a derivative of dimethylhydrazine, brings about a field change in the mucosa which makes it more susceptible to tumour formation. The subsequent development of tumours in areas of field change supports this theory. It would be difficult to prove directly that the adenomata are the forerunners of carcinomata, but a certain amount of circumstantial evidence exists in the DMH model system to suggest this is true. The carcinomata found were histologically similar to the adenomata except for the invasion of the former through the muscularis mucosa. Some of the adenomatous tumours which had not invaded had extremely anaplastic cells, typical of carcinomata. Carcinomata were found only in animals killed after the longest periods of treatment (30 weeks and more), indicating that they take longer to develop than adenomata and they were always found in areas which bore adenomatous polyps in animals treated for a shorter time.

Springer *et al.* (1970) described an increase in [$^{3}H$] TdR uptake in the mucosa as a precancerous change. The acute changes in proliferation demonstrated using [$^{125}I$] IUdR technique were accompanied by similar alterations of DNA synthesis in most organs in the body. This clearly indicates that DMH has a general toxic effect on the body which results in cell death followed by regenerative proliferation. Thus, although there may be specific changes in the colon related to carcinogenesis, these could be masked by the general response of the widespread reaction to the toxic properties of DMH. Assessment of cell proliferation using colchicine blockage gave variable results. Some tumours appeared to have a considerable mitotic activity, in others it was low and similar to the result of stathmokinetic tests made on patients with primary adenocarcinoma of the colon (Bottomley and Cooper, 1973). In trying to assess the proliferative activity in the " normal " bowel epithelium in the vicinity of the tumours, distortion of the crypts made it difficult to obtain sufficient longitudinal sections through the whole length of crypts in order to compare the proliferative activity with that of the control tissue. However, the colchicine blockade indicated that there was no major extension of the proliferative zone, and the lack of gross morphological change in the villi of the small intestine strongly suggested that the animals either compensate for the recurrent damage caused by the weekly injection of DMH or there may be some form of adaptation to the chronic toxicity.

It is clear that the main disadvantage of DMH as a method of raising colon tumours is its severe toxic effect on the liver. The general reaction of the liver has many features similar to that produced by nitrosamines (Svoboda and Higginson, 1968). Eosinopilic nuclear inclusions in liver cells have been described as occurring as a result of subclinical disease in untreated animals (Wilson, 1954) and as a result of feeding mice and rats with toxic substances such as thioacetamide (Kleinfield, Greider and Frajola, 1956), colchicine (Wessel, 1958) and with a methionine rich diet with added bentonite (Leduc and Wilson, 1959). It was the latter authors who first demonstrated conclusively that these inclusions were of cytoplasmic origin and our own findings entirely substantiate this view. As yet the effects of chronic DMH poisoning on the metabolism of this carcinogen are unknown. It is possible that these metabolites are then acted upon by bacteria in the colon (Druckrey, 1970). The finding of bacteria in the deeper part of crypts of experimental animals may be of some importance as it could produce metabolites of DMH in close proximity to the dividing cells of the deeper part of the crypts. However, it is equally possible that the presence of the bacteria in the depths of the crypts may be a reflection of the alteration of the quality of mucopolysaccharides produced by the goblet cells. A change in mucopolysaccharide is a possible explanation for the loss of the surface crypt pattern as defined by the alcian blue staining technique (Fig. 2).

In the model system with once weekly injections of DMH for 22 weeks and more described in this paper, the incidence of adenomatous polyps was 100% in treated animals and in a trial series of only 17 weekly injections, followed by a rest period of 6–13 weeks. Hence there is a time during the injection schedule after which the eventual occurrence of adenomatous polyps in the distal part of the colon will be inevitable. It can be seen that the model system is ideal for studying the changes from the normal state through various preneoplastic lesions, with the eventual development of a tumour, and the major target site for neoplastic change is relatively limited. Furthermore, the several points of similarity between the structure and growth pattern of the adenomatous polyps and adenocarcinomata and their counterparts in man suggest the model may have some value as a system for screening various chemotherapeutic agents to be used for the treatment of colorectal cancer.

Unfortunately, the severe hepatic disease might well interfere with the action of various chemotherapeutic drugs and might lead to misinterpretation of any screening procedure. It is therefore important to attempt to improve this model by adjusting the dose and timing of the DMH injections in order to retain its property of inducing neoplasms, while at the same time reducing the level of damage to the liver. A single dose of DMH failed to induce tumours in 16 male and 16 female mice killed at intervals up to 17 months. In animals given only 14 or 17 weekly injections of DMH then left for at least 8 weeks before killing, histologically there was probably considerable improvement in liver function but this has not been tested formally. However, the number of tumours per animal was reduced, so it would seem that when using DMH to induce colon cancer in this particular strain of mice the dose required to produce a high number of tumours in 100% of the animals is always accompanied by liver damage.

We wish to thank Miss C. Nutman and Mr S. Adamthwaite for their skilled technical assistance.

This work was supported by the Yorkshire Cancer Research Campaign.

### REFERENCES

BIBBECK, M. S. C. & DUKES, C. E. (1963) Electron Microscopy of Rectal Neoplasms. *Proc. R. Soc. Med.*, **56**, 793.

BOTTOMLEY, P. & COOPER, E. H. (1973) Cell Proliferation in Colonic Mucosa and Carcinoma of the Colon. *Proc. R. Soc. Med.* In the press.

DRUCKREY, H. (1970) Production of Colonic Carcinomas by 1,2-Dialkylhydrazines and Azoxyalkanes. In *Carcinoma of the Colon and Antecedent Epithelium*, Chapt. 20. Ed. W. J. Burdette. Illinois: Thomas.

DRUCKREY, H., PREUSSMAN, R., MATZKIES, F. & IVANKOVIC, S. (1967) Selektive Erzeugung von Darmkrebs bei Ratten durch 1,2-Dimethyl-Hydrazin. *Naturwissenschaften*, **54**, 285.

FILIPE, M. I. (1971) $^{35}$Sulphur Uptake in the Mucosa Adjacent to Carcinoma of the Large Intestine. *Histochem. J.*, **3**, 27.

FILIPE, M. I. (1972) The Value of a Study of the Mucosubstances in Rectal Biopsies from Patients with Carcinoma of the Rectum and Lower Sigmoid in the Diagnosis of Premalignant Mucosa. *J. clin. Path.*, **25**, 123.

FISHER, E. R. & SHARKEY, D. A. (1962) The Ultrastructure of Colonic Polyps and Cancer with Special Reference to the Epithelial Inclusion Bodies of Leuchtenberger. *Cancer, N.Y.*, **15**, 160.

IMAI, H. & STEIN, A. A. (1963) Ultrastructure of Adenocarcinoma of the Colon. *Gastroenterology*, **44**, 410.

IMAI, H., SAITO, S. & STEIN, A. A. (1965) Ultrastructure of Adenomatous Polyps and Villous Adenomas of the Large Intestine. *Gastroenterology*, **48**, 188.

KLEINFELD, R. G., GREIDER, N. H. & FRAJOLA, W. J. (1956) Electron Microscopy of Intranuclear Inclusions Found in Human and Rat Liver Parenchymal Cells. *J. biophys. biochem. Cytol.*, **2**, Suppl., 435.

KNOWLES, J. C., WEAVERS, B. & COOPER, E. H. (1972) Accumulation of Calcium in the Intramitochondrial Dense Bodies in Mice. *Exptl Cell Res.*, **73**, 230.

LANGVAD, E. (1968) Lactate Dehydrogenase Isoenzyme Patterns in Tumour Bearing Colon. *Int. J. Cancer*, **3**, 17.

LEDUC, E. H. & WILSON, J. W. (1959) An Electronic Microscopic Study of Intranuclear Inclusions in Mouse Liver and Hepatoma. *J. biophys. biochem. Cytol.*, **6**, 427.

LÖHRS, U., WIEBECKE, B. & EDER, M. (1969) Morphologische und autoradiographische Untersuchung der Darmschleimhautveranderungen nach einmaliger Injektion von 1,2-Dimethylhydrazin. *Z. Ges. exp. Med.*, **151**, 297.

PEARSE, A. G. E. (1968) *Histochemistry: Theoretical and Applied*, 3rd Edn., Vol. 1. London: J. A. Churchill Ltd.

PEGG, A. E. & HAWKS, A. (1971) Increased Transfer Ribonucleic Acid Methylase Activity in Tumours Induced in the Mouse Colon by the Administration of 1,2-Dimethylhydrazine. *Biochem. J.*, **122**, 121.

SCHAUER, A., VOLLNAGEL, T. & WILDANGER, F. (1969) Cancerisierung des Rattendarmes durch 1,2-Dimethylhydrazin. *Z. Ges. exp. Med.*, **150**, 87.

SPJUT, H. J. & SMITH, M. N. (1967) A Comparative Electron Microscopic Study of Human and Rat Colonic Polyps and Carcinomas. *Exp. molec. Path.*, **6**, 11.

SPRINGER, P., SPRINGER, J. & OEHLERT, W. (1970) Die Vorstufen des 1,2-Dimethylhydrazin-unduzierten Dick- und Dünndarmcarcinoms der Ratte. *Z. Krebsforsch.*, **74**, 236.

SVOBODA, D. & HIGGINSON, J. (1968) A Comparison of Ultrastructural Changes in Rat Liver due to Chemical Carcinogens. *Cancer Res.*, **28**, 1703.

TARIN, D. (1970) Assessment of the Significance of the Intramitochondrial Dense Body in Carcinogenesis. *J. invest. Derm.*, **55**, 26.

WEISBURGER, J. H. (1971) Colon Carcinogens: Their Metabolism and Mode of Action. *Cancer, N.Y.*, **28**, 60.

WESSEL, W. (1958) Elektronenmikroskopische Untersuchungen von intranuclearen Einschlusskörpern. *Virchows Arch. path. anat., Physiol.*, **331**, 314.

WIEBECKE, B., LOHRS, N., GIMMY, J. & EDER, M. (1969) Erzeugung von Darmtumoren bei Mausen Durch 1,2-Dimethylhydrazin. *Z. Ges. exp. Med.*, **149**, 277.

WILSON, J. W. (1954) Nuclear Inclusions in the Mouse Liver. *Anat. Rec.*, **118**, 368.